# Effect of different surgical routes on pregnancy outcome of history-indicated cervical cerclage

**DOI:** 10.1007/s00404-023-07007-7

**Published:** 2023-04-01

**Authors:** Feng Qin, Yong Yang, Wei Zhou, Yugang Chi, Bao Liu, Gongli Chen

**Affiliations:** Department of Gynaecology and Obstetrics, Chongqing Health Center for Women and Children, Women and Children’s Hospital of Chongqing Medical University, Chongqing, 401147 China

**Keywords:** Cervical incompetence, Cervical cerclage, Laparoscopy, Vaginal

## Abstract

**Objective:**

To study the guiding significance of medical history on laparoscopic and vaginal cervical cerclage in the treatment of cervical incompetence and its influence on pregnancy outcome.

**Methods:**

A total of 53 cases by laparoscopic abdominal cervical cerclage (LAC group) before pregnancy and 73 cases by transvaginal cervix cerclage (TVC group) at 12–14 weeks of pregnancy were collected. Multivariate logistic regression analysis was performed on the influencing factors of delivery gestational weeks. Furthermore, the gestational weeks after cervical cerclage were compared between the two groups with high- and low-risk grades.

**Results:**

The number of previous uterine cavity operations in LAC group was more than that TVC group, and the costs of operation were more than TVC group. At the same time, the hospitalization days and operation time were longer than those in TVC group, and the delivery rate of cesarean section was higher than TVC group, but the total hospitalization times were less than TVC group (*P* < 0.05). The rate of delivery before 34 weeks of pregnancy and the incidence of premature rupture of membranes or premature labor in LAC group were lower than those in TVC group (*P* < 0.05). In TVC group, the increased number of prior PTB or STL and the history of cervical cerclage failure would increase the risk of premature delivery before 34 weeks of pregnancy. There was no increased risk of preterm delivery before 34 weeks of pregnancy in LAC group (*P* > 0.05). According to the risk level, in the high-risk group, the delivery rate of LAC group at gestational weeks < 37 weeks, < 34 weeks and < 28 weeks was lower than that of TVC group.

**Conclusion:**

Laparoscopic cervical cerclage might be more effective in preventing premature delivery before 34 weeks of gestation, and its influence on delivery gestational weeks was not affected by related medical history. For high-risk patients with the history of prior PTB or STL and failed cerclage, laparoscopic cervical cerclage might be more effective than vaginal cervical cerclage in preventing extremely preterm before 28 weeks, premature delivery before 34 weeks and premature delivery before 37 weeks. Therefore, our limited experience suggested that LAC can be a recommended option for patients with high-risk history.

## What does this study add to the clinical work

**Table:** 

Analyzing the effect of different surgical routes can help to choose the optimal treatment and provide guidance for optimal preoperative planning in the clinical work, improve the prognosis, and reduce the perinatal morbidity. At the same time, this study also has guiding significance for the management and treatment of pregnant women after cervical cerclage.	

## Introduction

Cervical incompetence (CIC) is due to the abnormal anatomical structure or function of the cervix, which leads to the progressive and painless shortening, flattening, and expansion of the cervix before the 37th week of pregnancy, with or without premature rupture of membranes and protruding amniotic sac at the cervix, and can easily cause the inability to maintain the pregnancy in the second and third trimesters of pregnancy. The incidence of CIC in premature delivery is 0.1%–1.0% [1], and it accounts for 15% of recurrent spontaneous abortion in 16–28 weeks of pregnancy [2]. At present, the etiology of CIC is not completely clear [3], mainly including recurrent second-trimester pregnancy abortion or premature delivery, history of cervical trauma, infection factors, congenital cervical dysplasia or uterine malformation [4], etc. CIC can lead to an increase in perinatal morbidity, including premature rupture of membranes, chorioamnionitis and premature delivery [5]. Therefore, it is of great significance to effectively evaluate the treatment methods and related factors of CIC to prevent premature delivery and improve the reproductive quality.

At present, cervical cerclage is the main surgical method to treat CIC. According to the guidelines of Royal College of Obstetricians and Gynaecologists (RCOG) in 2022 [6], cervical cerclage can be divided into cervical cerclage indicated by medical history, cervical cerclage indicated by ultrasound, and cervical cerclage indicated by physical examination. Among them, preventive cervical cerclage with medical history indication is an effective method to treat CIC [7]. Indicative preventive cervical cerclage can be performed via vagina or abdomen. McDonald's and Shirodkar's methods are two main methods of transvaginal cerclage [8]. Transvaginal operation is convenient and easy to operate and has been used in clinical practice for over 60 years. However, transvaginal cerclage may be technically limited in many cases such as excessive cervical position or other anatomic abnormalities [9]. Transabdominal cerclage may be another effective way, while laparoscopy has the advantages of minimally invasive, avoiding foreign bodies in vagina and infection [10, 11]. However, cesarean section is the main way to terminate pregnancy for women who underwent laparoscopy, which increases the surgical complications. Analyzing the effect of different surgical routes can help to choose the optimal treatment, improve the prognosis, and reduce the perinatal morbidity. This article retrospectively analyzes the differences between laparoscopic and vaginal preventive cervical cerclage, compares the effects of the two surgical methods on the gestational age of delivery, and explores the potential influencing factors related to the two surgical methods, providing guidance for optimal preoperative planning.

## Patients and methods

### Study population

This retrospective research was carried out in the Department of Gynaecology and Obstetrics, Chongqing Health Center for Women and Children, Chongqing, China. We collected a total of 216 patients with indications of preventive cervical cerclage due to cervical incompetence between January 2018 and December 2021, which including 98 patients with laparoscopic cervical cerclage and 118 patients with transvaginal cervical cerclage. The medical history, clinical data and pregnancy outcomes were retrospectively reviewed. Laparoscopic cervical cerclage was pre-pregnancy cervical cerclage, among which 45 patients were excluded without pregnancy outcome by the end of follow-up, including 18 cases lost to follow-up, 12 cases without pregnancy plan, 9 cases currently undergoing assisted reproduction, and 6 cases in pregnancy; 26 patients with transvaginal cerclage lost follow-up, and 19 twins were excluded from this study. Finally, this study included 53 cases of cervical cerclage with pregnancy outcome by laparoscopy before pregnancy, and 73 cases of prophylactic cervical cerclage with pregnancy outcome by vagina at 12–14 weeks of pregnancy.

### Eligibility criteria

① One or more cases of abortion or premature delivery in the second trimester of pregnancy due to painless cervical dilatation; ② positive uterus enlargement test in non-pregnancy period; ③ patients and their families fully understand and sign the informed consent form; ④ the medical history was complete.

### Exclusion criteria

① Multiple pregnancy; ② termination of pregnancy and medical premature birth for medical reasons; ③ emergency ring ligation and emergency ring ligation; ④ signs of abdominal pain, infection, or bleeding; ⑤ patients with coagulation dysfunction.

### Surgical techniques

Urine examination, routine examination of vaginal secretion and general bacterial culture were performed in both groups before operation, all of which indicated that there was no infection of genitourinary organs. All the patients in the vaginal group underwent ultrasound examination to confirm the fetal survival before operation, and combining with serum chromosome and ultrasound examination to check the malformation. Both groups were well prepared before operation, and the contraindications of operation and anesthesia were excluded. Here, a unified Mersikence loop tie (polypropylene loop tie with needles at both ends, 40 cm in length and 5 mm in width) for cervical cerclage was used.

### Laparoscopic abdominal cervical cerclage

The specific operation of laparoscopic abdominal cervical cerclage (LAC) was as follows: after the patient was placed in supine position and given general anesthesia, laparoscopic perforation was performed on both sides of the umbilical region and lower abdomen. During the operation, the uterus was pushed forward by a uterine lifter, and the upper edge of the root of bilateral sacral ligaments was taken about 1 cm. The inner edge of uterine artery was the puncture point, and the polypropylene loop was punctured. Four knots were tied on the front wall of cervical canal. Finally, there was no abnormality in the uterine cavity after hysteroscopy. Antibiotics were routinely used to prevent infection after operation. One month after operation, the ladies began to actively try to get pregnancy. The suture should be removed by cesarean section from normal pregnancy to the third trimester of pregnancy.

### Transvaginal cervix cerclage

The specific operation of transvaginal cervix cerclage (TVC) was as follows: the pregnant woman was performed continuous epidural anesthesia. We took the loop strap, inserted the needle at 11 o'clock, and then took out the needle at 10 o′ clock for purse-string suture. At the last 2 o′ clock, the needle was inserted at 1 o′ clock, avoiding the vascular plexus, and the suture depth reached 2/3 of the cervical muscularis, without penetrating the mucosa. The loop strap was gradually tightened at the anterior fornix. Antibiotics were routinely used to prevent infection and desmoprogesterone 10 mg q8h was treated after operation. The suture can be removed at 36–38 weeks of pregnancy from normal pregnancy to the third trimester of pregnancy. If the patient had the premature labor, the cervical cerclage line was removed immediately.

### Date collection

Date were collected from the patients’ medical records and telephone follow-up. We reviewed the subjects’ medical history, and collected general clinical features, surgical conditions, pregnancy outcomes, etc. We defined the risk level, less than three prior PTB or STL (PTB: preterm birth; STL: second-trimester loss) and no history of cervical cerclage failure as low-risk group (35 cases in LAC group and 50 cases in TVC group), three or more prior PTB or STL (8 cases in LAC group and 10 cases in TVC group) and/or the history of cervical cerclage failure (16 cases in LAC group and 15 cases in TVC group) as high-risk group (18 cases in LAC group and 20 cases in TVC group).

### Statistical analysis

The data that conformed to the normal distribution were expressed as mean ± SD, and the comparison among the means was made by Student’s *T* test. The data that did not conform to the normal distribution was expressed as the median (interquartile range), M(P25, P75). The counting data were expressed by the number of cases (*n*) and percentage (%), and the comparison was made by Chi-square test or Fisher’s exact test. The factor analysis of factors related to gestational age of delivery was carried out by multivariate logistics regression model. A *P* value < 0.05 was considered significant. SPSS25 statistical software was used to analyze the data.

## Result

Comparison of clinical characteristics, and surgical conditions between LAC group and TVC group is shown in Table 1. There was no significant difference between the two groups in age, BMI, pregnancy times, parity, abortion or premature delivery times in the second trimester, history of previous cervical cerclage failure, history of cervical surgery, cervical length measured by B-ultrasound before pregnancy, and bleeding volume (*P* > 0.05). The number of uterine cavity operations between two groups was statistically significant (*P* < 0.05). The hospitalization days and operation time of LAC group were longer than those of TVC group, and the costs of operation were more than TVC group, while the total hospitalization times from pregnancy to delivery were less than those of TVC group (*P* < 0.05).

**Table 1:** Comparison of general clinical data and pregnancy outcome between the two groups

Characteristics	LAC group (*n* = 53)	TVC group (*n* = 73)	t/*x*^2^/z	*P* value
Age^a^ (years)	30.6 ± 3.8	29.6 ± 4.5	1.296	0.197
BMI^b^ (kg/m^2^)	23.53 (21.4, 25.5)	22.26 (20.2, 24.9)	1.735	0.083
Gestity^b^	3 (2, 4)	3 (2,4)	0.320	0.749
Parity^b^	0 (0, 0)	0 (0, 1)	1.624	0.104
Prior PTB or STL^c^				
≤ 1	29 (54.7%)	40 (54.8%)	0.059	0.971
2	16 (30.2%)	23 (31.5%)		
≥ 3	8 (15.1%)	10 (13.7%)		
Prior cerclage failure^c^				
No	37 (69.8%)	58 (79.5%)	1.539	0.215
Yes	16 (30.2%)	15 (20.5%)		
Prior cervical surgery^c^				
No	46 (86.8%)	62 (84.9%)	0.087	0.803
Yes	7 (13.2%)	11 (15.1%)		
Intrauterine operation^c^				
≤ 1	34 (64.2%)	64 (87.7%)	10.647	0.005
2	9 (17.0%)	6 (8.2%)		
≥ 3	10 (18.8%)	3 (4.1%)		
Cervical length before pregnancy^b^ (cm)	2.7 (2.5–3.0)	3.0 (2.5–3.3)	1.474	0.140
Hospitalization days^b^	7 (6, 8)	3 (3, 5)	0.397	< 0.001
Time of operation^b^ (min)	48 (45, 50)	20 (19.5, 34.5)	7.639	< 0.001
Amount of bleeding^b^ (ml)	10 (10, 20)	10 (5, 20)	1.868	0.062
Organ injury	0	0	–	–
Hospitalization times^b^	2 (2, 2)	2 (2, 2.5)	3.523	< 0.001
Costs of operation^a^ (RMB)	9340.3 ± 1244.7	4124.2 ± 1000.7	25.960	< 0.001

*BMI* body mass index, *PTB or STL* preterm birth or second-trimester loss

^a^$$\overline{x}$$ ± s

^b^M(P25, P75)

^c^*n*(%)

Comparison of pregnancy outcome and pregnancy complication between LAC group and TVC group is shown in Table 2. Compared with the mode of delivery, there were 50 cases (94.3%) of cesarean section in LAC group and 16 cases (21.9%) in TVC group. The rate of cesarean section in LAC group was higher than that in TVC group (OR 0.017, 95% CI 0.005–0.061, *P* < 0.05). The gestational weeks were divided into three groups according to 37 weeks, 34 weeks, and 28 weeks, respectively. There were 5 cases (9.4%) in LAC group and 18 cases (24.7%) in TVC group with gestational weeks less than 34 weeks. The rate of delivery before 34 weeks in LAC group was lower than that in TVC group (*P* < 0.05). 10 cases (18.9%) of newborns weighed less than 2500 g in LAC group, and 26 cases (35.6%) in TVC group, which were lower than those in LAC group (OR 0.420 and 95%CI 0.182–0.972, *P* < 0.05). That is, compared with the TVC group, LAC group had a better improvement effect on premature delivery and low birth weight. There was no significant difference in the rate of neonatal admission to intensive-care unit (NICU) between the two groups. There was no significant difference between the two groups in the first and second trimesters’ losses, and the complication such as breech presentation, placenta previa, hypertensive disorders complicating pregnancy, GDM, or ICP.

**Table 2:** Comparison of pregnancy outcome and pregnancy complication between the two groups [*n*(%)]

Outcome and complication	LAC group (*n* = 53)	TVC group (*n* = 73)	$x$^2^	*P* value
Delivery				
Cesarean	50 (94.3%)	16 (21.9%)	64.567	< 0.001
Vaginal	3 (5.7%)	57 (78.1)		
Delivery gestational age (weeks)				
< 37	13 (24.5%)	29 (39.7%)	3.192	0.074
≥ 37	40 (75.5%)	44 (60.3%)		
< 34	5 (9.4%)	18 (24.7%)	4.769	0.029
≥ 34	48 (90.6%)	55 (75.3%)		
< 28	5 (9.4%)	14 (19.2%)	2.277	0.131
≥ 28	48 (90.6%)	59 (80.8%)		
Neonatal weight (g)				
< 2500	10 (18.9%)	26 (35.6%)	4.221	0.040
≥ 2500	43 (81.1%)	47 (64.4%)		
NICU	4 (7.5%)	10 (13.7%)	1.176	0.278
First and second trimesters losses	5 (9.4%)	14 (19.2%)	2.277	0.131
PPROM/premature	10 (18.9%)	27 (37%)	4.860	0.027
Breech presentation	5 (9.4%)	5 (6.8%)	0.038	0.845
Placenta previa	1 (1.9%)	0		0.421
Hypertensive disorders complicating pregnancy	5 (9.4%)	4 (5.5%)	0.251	0.617
GDM or ICP	7 (13.2%)	13 (17.8%)	0.487	0.485
Adhesion of loop tie	1 (1.9%)	0		0.421
Fracture or dislocation of the loop tie	0	1 (1.4%)		1.000
Retained of the loop tie	7	0	–	–

*NICU* neonatal intensive-care unit, *PPROM* preterm premature rupture of the membranes, *GDM* gestational diabetes mellitus, *ICP* intrahepatic cholestasis of pregnancy

Multivariate logistic regression analysis was performed on the influencing factors of delivery gestational age (Table 3), with delivery gestational age as dependent variable, ≥ 34 weeks as 0 and < 34 weeks as 1, and age, BMI, operation mode, prior PTB or STL, prior cervical cerclage failure, prior cervical surgery, and prior uterine cavity operation as independent variables. The results showed that different surgical routs had statistically significant effects on delivery gestational weeks (OR = 5.625, 95% CI 1.504–21.032, *P* < 0.05). The history of prior PTB or STL had statistical significance on the gestational weeks of delivery (OR = 2.755, 95% CI 1.406–5.398, *P* < 0.05). The increase of the number of prior PTB or STL would increase the risk of premature delivery before 34 week gestation. The history of cervical cerclage failure had statistical significance on the gestational age of delivery (OR = 3.682, 95% CI 1.206–11.243, *P* < 0.05), and would increase the risk of delivery before 34 weeks of pregnancy. The history of cervical surgery, the number of uterine cavity operations, age, and BMI had no statistical significance on delivery before 34 week gestation.

**Table 3:** Multivariate logistic regression analysis of the factors affecting labor gestational age (34 weeks) in both groups

Variable	Group	*β*	SE	Wald	*P* value	OR	95% CI
Age (years)		0.035	0.062	0.317	0.573	1.036	0.917–1.170
BMI (kg/m^2^)		− 0.082	0.084	0.949	0.330	0.921	0.781–1.086
Surgical routes	LAC^d^	− 1.727	0.673	6.588	0.010	5.625	1.504–21.032
	TVC						
Prior PTB or STL	≤ 1^d^	1.013	0.343	8.712	0.003	2.755	1.406–5.398
	2						
	≥ 3						
Prior cerclage failure	No^d^	1.303	0.570	5.237	0.022	3.682	1.206–11.243
	Yes						
Prior cervical surgery	No^d^	− 0.461	0.732	0.396	0.529	0.631	0.150–2.649
	Yes						
Intrauterine operation	≤ 1^d^	0.416	0.420	0.980	0.322	1.515	0.665–3.452
	2						
	≥ 3						

^d^Control group

Multivariate logistic regression analysis was performed on the gestational weeks of delivery in LAC group and TVC group, respectively (Table 4), and the differences of influencing factors between the two groups were compared. The results showed that the number of prior PTB or STL and the history of prior cerclage failure in the TVC group had statistical significance on the gestational weeks. With the increasing of the number of prior PTB or STL would increase the risk of premature delivery before 34 weeks after vaginal cerclage (OR = 3.050, 95% CI 1.352–6.879, *P* < 0.05). The failure history of cervical cerclage would increase the risk of delivery before 34 weeks after vaginal cerclage (OR = 6.270, 95% CI 1.680–23.399, *P* < 0.05). In the LAC group, the number of prior PTB or STL, as well as the history of cervical cerclage failure had no significant influence on the delivery before 34 weeks of pregnancy after laparoscopic cerclage.

**Table 4:** Multivariate logistic regression analysis of influencing factors of gestational age in both groups

	Variable	Group	*β*	SE	Wald	*P* value	OR	95% CI
LAC	Prior PTB or STL	≤ 1^e^	1.047	0.625	2.811	0.094	2.850	0.838–9.695
		2						
		≥ 3						
	Prior cerclage failure	No^e^	− 1.496	1.250	1.432	0.231	0.224	0.019–2.597
		Yes						
TVC	Prior PTB or STL	≤ 1^e^	1.115	0.415	7.223	0.007	3.050	1.352–6.879
		2						
		≥ 3						
	Prior cerclage failure	No^e^	1.836	0.672	7.465	0.006	6.270	1.680–23.399
		Yes						

^e^Control group

Furthermore, we included the patients with less than three prior PTB or STL and no history of cervical cerclage failure in the low-risk group (35 cases in laparoscopic group and 50 cases in vaginal group), and the patients with three or more prior PTB or STL and/or history of cervical cerclage failure were in the high-risk group (18 cases in LAC group and 23 cases in TVC group), among which the patients with more prior PTB or STL and history of cervical cerclage failure were in LAC group (Table 5). In the LAC group, the mean gestational age at delivery was lower in the high-risk group than in the low-risk group [37.3 (35, 38) vs. 38 (37.3, 38.1), OR 0.5, 95% CI 0.0–1.20, *P* < 0.05]. In the low-risk group, there was no significant difference between the LAC group and the TVC group in the gestational weeks of delivery < 37 weeks, < 34 weeks and  < 28 weeks respectively. In the high-risk group, the LAC group had lower gestational weeks  < 37 weeks,  < 34 weeks, and  < 28 weeks, respectively, than TVC group [gestational weeks < 37 weeks 6 (33.3%) vs. 16 (69.6%), OR 0.219, 95% CI 0.058–0.821, *P* < 0.05, Gestational week < 34 weeks 2 (11.1%) vs. 11 (47.8%), OR 7.333, 95% CI 1.364–39.438, *P* < 0.05, gestational week < 28 weeks 2 (11.1%) vs. 9 (39.1%), OR 5.143, 95% CI 0.947–27.921, *P* < 0.05].

**Table 5:** Analysis of delivery outcomes after cervical cerclage at different risk levels

	Gestational weeks	LAC (*n*, %)	TVC (*n*, %)	$x$^2^	*P* value
Low-risk group	< 37 w	5 (14.3)	14 (28.0)	2.231	0.135
	< 34 w	2 (5.7)	7 (14.0)	1.493	0.222
	< 28 w	2 (5.7)	5 (10.0)	0.500	0.479
High-risk group	< 37 w	6 (33.3)	16 (69.6)	5.331	0.021
	< 34 w	2 (11.1)	11 (47.8)	6.286	0.012
	< 28 w	2 (11.1)	9 (39.1)	4.038	0.044

## Discussion

Cervical incompetence is one of the main causes of pregnancy loss. Successful cervical cerclage will improve the outcome of pregnancy, prolong the pregnancy as much as possible, and reduce the cost of treatment and rehabilitation of premature infants [12]. It is safe and feasible to perform laparoscopic cervical cerclage with medical history indication in non-pregnant women with cervical incompetence, plus the inherent advantages of minimally invasive surgery, and it has better obstetric outcomes [13]. In this paper, we retrospectively studied the influence of laparoscopic and transvaginal surgical routes on pregnancy outcome in view of the indication of cervical cerclage with medical history. It showed that laparoscopic cervical cerclage was better than vaginal cervical cerclage in preventing premature delivery before 34 weeks of pregnancy, and superior to vaginal cervical cerclage in newborn weight. In one recent research report, Shennan et al. [14] pointed out that abdominal cerclage was recommended for cervical incompetence patients with a history of abortion in the second trimester of pregnancy, compared with transvaginal cervical cerclage, and abdominal cerclage significantly reduces premature delivery before 32 weeks. The results were consistent with previous studies. Moawad et al. [15] had pointed out that transabdominal cerclage can significantly reduce the premature delivery rate before 34 weeks. It was suggested that laparoscopic surgery was an effective intervention to sustain pregnancy to the stage of viability [16]. Tian et al. [7] found that the number of babies delivered at ≥ 34 weeks in the laparoscopic group was significantly higher compared with the transvaginal group.

The timing of laparoscopic cervical cerclage surgery is mostly before pregnancy, and some of them are performed at 6–8 weeks of pregnancy, because the small uterus is more suitable for laparoscopic surgery at this time. Transvaginal cerclage is usually performed in 12–14 weeks during pregnancy when the fetus is stable, which reduces the impact of surgical stimulation on the fetus. In this paper, laparoscopic cervical cerclage before pregnancy and vaginal cerclage during pregnancy were selected. Both groups were preventive cerclage, which excluded threatened abortion such as abdominal pain and vaginal bleeding, and avoided the influence of operation timing on the research outcome. Previous studies had pointed out that preoperative cervical length [17], age, BMI, prior PTB, or STL [18] were independent risk factors that affect pregnancy outcome. In our study, there was no significant difference in age, BMI, prior PTB or STL, prior cervical cerclage failures, cervical operations, and the length of cervix in non-pregnancy between the two groups. The inclusion of the two groups was reasonable. The number of uterine cavity operations in LAC group was more than that in TVC group, which was related to hysteroscopy before laparoscopic cervical cerclage, so as to ensure the good condition of uterine cavity and eliminate intrauterine adhesion. Here, the number of uterine cavity operations was not the influencing factor of delivery gestational age, while Gokce et al. [19] proposed that the full-term delivery rate of women who underwent hysteroscopy within 6 months before pregnancy decreased significantly. The difference between this study and the literature was related to the fact that this study focuses on the number of uterine cavity operations, and did not group the specific time of uterine cavity operations.

In this study, different route of cervical cerclage operation, the number of prior PTB or STL, and the history of cervical cerclage failure were all independent influencing factors of preterm birth before 34 weeks of gestation. The increase of prior PTB or STL and the history of cervical cerclage failure would increase the risk of premature delivery before 34 weeks after vaginal cerclage. However, prior PTB or STL and the history of cervical cerclage failure had no significant effect on preterm delivery before 34 weeks of pregnancy after laparoscopic cervical cerclage. Therefore, we believe that laparoscopic cervical cerclage is more effective in improving early and middle term preterm delivery, and the effect on gestational age at delivery is not easily affected by medical history-related factors. It is related to the following points: (1) laparoscopic cervical cerclage is higher than vaginal cerclage, which is closer to the cervical isthmus, and the cervical length does not change during the whole pregnancy [7, 20]. (2) Laparoscopic cervical cerclage is pre-pregnancy cerclage, which avoids the adverse effects of stimulation of cervical surgery during pregnancy on pregnancy [21]. (3) The infection risk of transvaginal surgery is higher than that of abdominal surgery. (4) Cervical dysfunction caused by abortion or premature delivery in the second trimester is related to chronic endometritis [22]. Laparoscopic cervical cerclage before pregnancy is beneficial to improve the microecology of endometrium before pregnancy and facilitate embryo implantation and development.

At present, it is recommended that preventive cervical cerclage is feasible for patients with three or more spontaneous preterm births or miscarriages [6], and there is no conclusion on the treatment of patients with less than three trimester miscarriages or preterm births. Therefore, it is very important to identify the high-risk patients with cervical incompetence early and accurately, and then take active intervention measures to improve the pregnancy outcome. Huang et al. [23] proposed that laparoscopic cervical cerclage was an effective method to treat cervical incompetence with failed transvaginal cerclage. This study showed that a history of three or more prior PTB or STL and cervical cerclage failure was a high-risk factor of cervical insufficiency, and can influence the gestational age of delivery after TVC. Moreover, LAC was more effective than TVC in preventing extremely preterm before 28 weeks, premature delivery before 34 weeks and premature delivery before 37 weeks. Therefore, LAC could be preferred for patients with high-risk history, especially with previous cervical cerclage failure and three or more prior PTB or STL. LAC was also recommended for patients with very short or absent cervix and severe cervical scars that cause difficulty in transvaginal cervix [24]. For low-risk people, TVC should still be tried first. For patients with spontaneous abortion history, uterine cervical length can be monitored by ultrasound during pregnancy [23], and preventive cervical cerclage can be performed when necessary.

One of the drawbacks of LAC is the termination of pregnancy by cesarean section in the third trimester. In this study, the rate of cesarean section in the LAC group was higher than that in the TVC group. Since the cesarean section rate is affected by different regions, institutions, pregnant women, and other factors; therefore, whether laparoscopic cervical cerclage increases the cesarean section rate needs to be studied with larger sample and multi-center. Although cesarean section in the third trimester has related complications for mothers and children [25–27], the benefits outweigh the disadvantages for women who have multiple miscarriages and fail to maintain pregnancy normally. In addition, some women will choose to keep the loop tie to guarantee the next pregnancy. In this study, seven cases chose to keep the tie, and there was normal menstrual flow and no discomfort during the follow-up. Ades et al. [28] analyzed 22 women who underwent laparoscopic cervical cerclage and retained cervical cerclage in situ. Among them, 19 cases gave birth after two pregnancies, and 3 cases gave birth three times. Their third pregnancy was as successful as the first and second pregnancies. In the LAC group, there were 3 cases of non-cesarean section delivery, included one with 8 weeks of pregnancy missed abortion through dilation and evacuation, and the other two cases were inevitable miscarriages at 20 weeks and 22^+2^ weeks who were delivered vaginally after laparoscopic removal of the loop tie. Therefore, vaginal delivery after removal of the loop tie by laparoscopic may be a potential choice for first and second trimesters’ losses. At the follow-up of this study, there were 9 patients with artificial assisted reproduction. The specific reasons of infertility were mostly ovarian dysfunction, male factors and fallopian tube factors, and there was no evidence related to laparoscopic cervical ligation surger, so we agreed with Demirel et al. [16] who believed that LAC was a very effective intervention to sustain pregnancy to the stage of viability, which did not delay achieving pregnancy and did not have a negative impact on the chances of conception.

The study followed up two groups of pregnancy complications after cerclage. There was no significant difference between the two groups for the complication such as breech presentation, placenta previa, hypertensive disorders complicating pregnancy, GDM, or ICP in first and second trimesters losses. Therefore, it was believed that LAC did not increase the occurrence of pregnancy complications during pregnancy compared with TVC. Gynecologists were required to have rich experience in minimally invasive surgery for the LAC. At the same time, due to the strict requirements of laparoscopic transabdominal surgery, general anesthesia and fasting, as well as abdominal incision healing and other factors, the hospitalization days and operation time of LAC were longer than those of TVC and the costs of operation were more than TVC group. However, the total number of hospitalizations was reduced, which avoids the infection of vaginal operation, absolute bed rest, and fetus protection rate in hospital. It was worth mentioning that there were some perioperative complications after cervix cerclage [29], and LAC had the risk of adhesion and exposure. In this study, there was one case in which the adhesion of the loop tie was difficult to remove during cesarean section, without erosion of the peripheral viscera.

There were several limitations in our study. First, due to the small number of cases and related studies, there was no strong evidence to show the pregnancy outcome of retaining laparoscopic cervical girdle in situ, which needs to be continuously observed and followed up. Second, this was a retrospective study. Although laparoscopic surgery was performed by only one doctor who had surgical qualifications and rich experience, the survey might still be affected by the choice of surgeons. Therefore, prospective study is required to further validate the influence of surgical routes on pregnancy outcome.

## Conclusion

Laparoscopic cervical cerclage might be more effective in preventing premature delivery before 34 weeks’ gestation, and the influence of gestational weeks was not affected by the number of prior PTB or STL, as well as the history of cervical cerclage failure. The number of prior PTB or STL and the history of cervical cerclage failure were independent factors influencing the gestational age of delivery after TVC, which would increase the risk of premature delivery before 34 weeks of pregnancy. In high-risk patients with the history of prior PTB or STL and failed cerclage, LAC might be more effective than vaginal cerclage in preventing abortion before 28 weeks, premature delivery before 34 weeks, and premature delivery before 37 weeks. Therefore, our limited experience suggested that LAC can be a recommended option for patients with high-risk history, whereas TVC can still be tried first for low-risk people. However, laparoscopic cervical cerclage tends to increase cesarean section rate compared with vaginal cervical cerclage, and the advantages and disadvantages of retaining laparoscopic cervical cerclage in situ still need further follow-up and research.

## Data Availability

The data that support the findings of this study are available from the corresponding author, Gongli Chen, upon reasonable request.
